# A New Perspective to Interpret How the Vestibular Efferent System Correlates the Complexity of Routine Balance Maintenance with Management of Emergency Fall Prevention Strategies

**DOI:** 10.3390/audiolres14030044

**Published:** 2024-06-18

**Authors:** Neil S. Longridge, Arthur I. Mallinson

**Affiliations:** Division of Otolaryngology, Department of Surgery, Faculty of Medicine, University of British Columbia, Vancouver, BC V5Z 1M9, Canada

**Keywords:** otoliths, utricle saccule, hair cells, vestibular efferents, ambulation feedforward evolution, vestibular morphology

## Abstract

Bipedalism is unique among mammals. Until modern times, a fall and resulting leg fracture could be fatal. Balance maintenance after a destabilizing event requires instantaneous decision making. The vestibular system plays an essential role in this process, initiating an emergency response. The afferent otolithic neural response is the first directionally oriented information to reach the cortex, and it can then be used to initiate an appropriate protective response. Some vestibular efferent axons feed directly into type I vestibular hair cells. This allows for rapid vestibular feedback via the striated organelle (STO), which has been largely ignored in most texts. We propose that this structure is essential in emergency fall prevention, and also that the system of sensory detection and resultant motor response works by having efferent movement information simultaneously transmitted to the maculae with the movement commands. This results in the otolithic membrane positioning itself precisely for the planned movement, and any error is due to an unexpected external cause. Error is fed back via the vestibular afferent system. The efferent system causes macular otolithic membrane movement through the STO, which occurs simultaneously with the initiating motor command. As a result, no vestibular afferent activity occurs unless an error must be dealt with.

## 1. Introduction

The transition to bipedal gait as a normal function, rather than as an extraordinary activity, occurred millions of years ago. A recently discovered battered fossil skeleton belonging to a human predecessor was determined to be 7 million years old. Many features of its femur (such as the twist in its shaft) suggested that this human relative walked on two feet. Studies of the angle of the skull on the spine also supported this suggestion [1]. In brief, bipedal gait set the stage for many evolutionary changes in human lineage [2].

During this process of development, there were also substantial changes in the bony structure of the vestibular temporal bone [3]. The semicircular canal dimensions in crania from southern Africa attributed to Australopithecus and Paranthropus resemble those of the great apes. However, the canal dimensions of Homo erectus, the earliest species to demonstrate modern human morphology (i.e., upright posture and obligatory bipedalism), are unlike those seen in any of the early hominids, but are similar to those of the modern human [3]. A very recent cadaver study in humans [4] demonstrated the close interrelations of the membranous labyrinth with the surrounding temporal bone. While the changes in the soft tissue structures of the inner ear are not known, it is probable, based on structural changes in the bone, that there were also substantial soft tissue changes in the balance system and in the mechanics of inner ear function during the evolution from quadrupedal to bipedal locomotion.

## 2. Clinical Relevance of Animal Experiments

As clinicians, the authors are most interested in balance system function in humans. Understanding its complexity is a huge challenge, and even today it is very poorly understood. For example, one can see the need to encode head motion in a context-independent manner during natural behaviours. Investigation of basic vestibular structure and function has largely been carried out on lower animals. The efferent vestibular system in the rat has been studied extensively [5]. Previous studies in macaques have come to differing conclusions (possibly because of difficulties studying how the vestibular system carries out trained movement, as opposed to natural spontaneous movement behaviour executed by humans). As a result, understanding of the mammalian vestibular efferent system in everyday life remains poor. This is well summarized in a very recent paper by Cullen and Wei [6], but extrapolating nonhuman function to humankind should be carried out with extreme caution. This present paper addresses human balance system function using our present knowledge, recognizing that routine bipedal gait is very different from ambulation in the quadruped and is almost unique in post marsupial mammalian species. The authors will discuss “emergency situations” with management of so-called “slip and fall” strategies, but also “fight or flight” reactions, both of which require sudden body stabilization.

## 3. Consequence of Differences between Quadrupedal and Bipedal Gait

Both bipeds and quadrupeds experience the impact effect of foot contact and resultant otoconial inertial movement when ambulating. During observation of a quadruped running (such as a dog), as opposed to a human, marked similarities, but also distinct differences, can be seen. The support system used by ambulating quadrupeds (using three points of contact) is far superior to that available to humans. A dog can run rapidly on highly uneven ground, including running along and over logs and across large crevasses, with no significant difficulty. A similar activity in a biped is slower, more cautious, and deliberate. The result of a single foot misplacement and subsequent insecurity in a human is potentially much more disastrous than a similar event in a four-footed animal.

Prior to hunter/gatherers establishing human settlement sites, walking was the standard means of perambulation, and running was utilized for speed in order to hunt or avoid predators. This latter activity requires perfect balance for evasion, and for survival. There are multiple examples of healed arm fractures in Neolithic graves; however, healed leg fractures are virtually unknown [7,8] (although survival following a surgical amputation has recently been documented in the late Pleistocene era, showing that some compassion for life-threatening injuries did exist in this era) [9]. In general, one can assume that in the bipedal hunter/gatherer, the consequence of a lower limb fracture would be devastating and probably fatal. This illustrates the need for a maximally effective fall prevention strategy.

## 4. Discussion of Normal Bipedal Gait and Emergency Management

During a normal gait cycle, the vestibular system regulates body orientation to maintain visual alignment; however, there is an increased weighting on vestibular macula during the heel strike in order to optimize foot placement planning [10]. The vestibular system must also be ready to cope with a sudden destabilization, such as a brief “slip and grip” of a foot on slippery ground; this is a familiar experience to most of us. How is this coped with in the biped? The result of a single foot misplacement and subsequent insecurity in a human is potentially much more disastrous than a similar event in a four-footed animal. While a quadruped still has a three-point stance available, a biped human must now bear weight on only one foot. This is much more challenging, in addition to the fact that a human has further to fall. Coping with this must include vestibular afferent input, as there is little time for a cerebral cortical/corticospinal response. This is because a forward trip can be counteracted by a rapid lift and movement of either the limb causing the trip or of the other limb (depending on the stage of the stepping process), and by the degree of resistance of the object causing the trip. This is learned by experience as a child. However, a slip event on an icy surface is also experienced when young, but the learned “contralateral limb lift” manoeuvre may not be as effective at preventing a fall under these conditions, as the slip may be sideways or backwards. Such a slip can be detected and coped with (if slow) by awareness that it has occurred and by making a rapid and appropriate corrective movement. However, if the event occurs very rapidly, on an icy surface, the feet may provide only minimal (possibly unreliable) information. Vision is not helpful because foveation, although very fast, cannot be accomplished and the resulting sensation of a “whirl of light” as the head moves is not useful. As a result, an extreme specialization of balance is required.

It is standardly taught that the balance system depends on integration of proprioceptive, visual, and vestibular afferent information. It is also clear that the balance maintenance system is not a single system but, rather, a complex integration of these three sets of sensory information and also a simultaneous analysis of the physical activity which is also occurring. These systems must work together to produce an appropriate response to any potentially destabilizing event. The basic balance maintenance processes we understand (in a system which has developed over millennia) are interspersed with extremely complex emergency management processes for critical balance situations. Such “emergency responses” that are sometimes required are difficult to conceive of, let alone investigate effectively. Another concern is that many of the impressive pioneering experiments carried out over the years used electrical stimulation of whole brainstem systems, which makes it difficult to interpret subtle responses.

Experimental evidence has shown that vestibular information is not reliably upregulated during unexpected destabilization events, such as an unexpected obstacle avoidance [10], and the role of the vestibular system during these events is unclear. The authors of this paper outline that “The context of greater uncertainty about obstacle positioning could result in a greater reliance on sensory, including vestibular information”. They also suggested that such events “benefits from a high level of planning” and foot placement at these times is directed by some “separate internal feedforward model”. We wondered where the “high level of planning” originated and what the location of the “internal feedforward model was.

The vestibulocollic reflex (VCR) is obviously part of the restabilizing process after a “slip-and-grip” event. The VCR is a compensatory response of the neck muscles when head motion is sensed by the vestibular organs in the inner ear. In addition, the cervicocollic reflex (CCR) will also be activated. The CCR is a compensatory response of the neck muscles driven by neck proprioceptive inputs during motion of the body. However, there must be a command signal to drive this response (i.e., a sudden change in plans to avoid an unexpected obstacle). We suggest that the otoconial system must be an essential part of this critical decision-making process (i.e., prior to any motor response), as a response (particularly in a biped) is required almost instantaneously. Basic neuroanatomy/neurophysiology outlined in textbooks details that vestibular information is the only information that reaches the cerebellum without synapsing, having its own dedicated route (a route through the inferior cerebellar peduncle known as the juxtarestiform body) into the cerebellum. Another mechanism which probably helps carry out these adjustments quickly and accurately is the so called “vestibular redundancy”. This is achieved by VIIIth nerve input into the pontine nuclei, which gives rise to transverse pontine fibres that become the middle cerebellar peduncle. (Anatomically, the pontine nuclei funnel into the middle peduncle to enter the cerebellum.) The pontocerebellar neurons provide mossy fibre input to the cerebellum. In other words, this is second-order vestibular information. This redundancy of information seen by the cerebellum may also aid in making the sudden adjustments that are required in an emergency situation. Initial vestibular analysis instructs the VCR and the CCR, in addition to informing the cerebral cortex so that an attempt can be made to prevent a fall, or at least try to minimize injury by putting out an elbow or protecting the head with a hand.

There is clinical evidence for participation of the vestibular system in the feedforward model. In vestibular patients, the rapid response system may be flawed and not rapid enough to avert a fall. In a patient with unilateral vestibular pathology, the normal functioning side acts precisely and consistently, as it has done since this skill was acquired, but the side with impaired function cannot produce the precise rapid response quickly enough (including VCR and CCR); these responses have all been learned from childhood falls. This asymmetrical emergency response results in a fall to the diseased side. It has been demonstrated that patients suffering a slip and fall and subsequent fracture usually sustain the fracture ipsilateral to any existing vestibular injury. This is true for both hip [11] and wrist fractures [12].

Pioneering studies by Goldberg and Fernandez [13] have suggested that a feedforward system with a rapid feedback mechanism must exist for critical balance maintenance, but such a system would be extremely difficult to evaluate from the basic science point of view, and the value of emergency response has to be viewed cautiously, especially in non-primates who may have very different requirements and needs. One purpose of our paper is to delineate a physiological and anatomical theory about how these systems may be integrated in bipedal humankind. We will specifically outline the role and significance of the striated organelle (STO) in this process, which will be discussed in detail later.

## 5. Relevance of the External Senses

Mammals have five senses through which they recognize their external environment. They are smell, taste, touch, vision, and hearing. In the English language, “intuition” is the sixth sense. The silent sense of balance is therefore the seventh sensory system, which humankind is often unaware of until it malfunctions. The balance system utilizes afferent visual and proprioceptive information. This information is combined with intrinsic vestibular information.

The vestibular system of the inner ear is an intrinsic gravity-dependent movement management system with three functions. In no particular order, these are as follows:

- To foveate; i.e., maintain eye fixation on a target during head movement. This is predominantly a semicircular canal task, although semicircular canal activation is modulated by the maculae of the utricle and saccule [14].
- To provide information regarding change of head position during movement of the neck or the body as a whole, during perambulation; this is a macular function.
- To provide a constant flow of information about the relationship of the head (and therefore the body) to gravity; this is also a macular function.

Each of the five external sensory systems uses an efferent response to an afferent stimulus, mediated through the cerebral cortex, with threat being the most alarming. The reflex response to a potential threat is a rapid head and/or body movement, for example, to a smell of burning, a taste related to food being “off”, or turning the head on hearing an unexpected sound. However, a soft touch in terms of a neighbourly pat or a specific prod can also be associated with a rapid head turn and perhaps a related body movement, (note that these actions require refoveation and restabilization).

We have all been in a situation while driving, with our thoughts on something else, when a sudden peripheral visual stimulus, such as an unexpected pedestrian or rapidly moving car, causes an extreme startle reaction, head turn, and, if needed, a response of rapid braking. Any of these responses require immediate movement of the head in order to foveate the object of concern. As discussed previously, this “emergency response system” is one in which the vestibular system plays a major role. Responses to daily postural perturbations occur more quickly than the fastest voluntary movements. Although the earliest postural responses do not involve a cortical loop, it is strongly suggested that the cerebral cortex is involved in shaping the postural response as it progresses. This influence is probably mediated directly, via corticospinal loops, and modified indirectly, via communication with brainstem centres. This provides both speed and flexibility for preselecting an environmentally appropriate response [15].

One of the authors (NSL) underwent a physical experience which would be in keeping with this. Because of his specialized clinical vestibular knowledge, he realized afterwards it was an example of these responses in action, and this experience was the instigation of the thought processes which have resulted in a proposal for a new and more complex concept of how the vestibular system interacts through the cerebral cortex, particularly with the proprioceptive aspect of balance maintenance during body movement. The author was fielding at a game of cricket at Cowichan on Vancouver Island and running for a ball on a manicured outfield that was short and flat. His foot went into a 2-ft. square of grass that was 2 inches below the level of the surrounding ground, but not visible, as all the grass was cut to the same length. The timed foot/ground contact predicted by the usual running process with flexor contraction was mistimed and resulted in a rapid unresisted leg flexion, with muscle spindle action cutting in, to reduce leg muscle damage. The later-than-expected foot/ground contact was associated with a lurch forward before the foot hit the ground. On later reflection he realized that this sensation of a lurch was due to mistimed velocity change: a lack of acceleration, due to a sudden unexpected alteration in the precisely timed foot contact. This lurch (the result of the unpredicted deceleration) was necessitated by the sudden unexpected alteration in predicted rhythmic macular otoconial movement during the running process. Further thought has resulted in the hypothesis we propose in this paper.

With the five external senses, afferent information elicits an efferent response. However, the balance system of the inner ear, an intrinsic system, works in a different fashion the “afferent-to-efferent” process utilized by the other sensory systems is not the only process it utilizes. We propose that in the vestibular system, the macular efferent system does not act in response to afferent information (from the vestibular system or from other sources), but that vestibular efferent axonal activity is a preplanned part of any head or body movement and results in an otolithic afferent vestibular “error detection” response. The error detection occurs simultaneously with peripheral muscle movement during any movement. Along with the “afferent to efferent” vestibular signal, an “efferent to afferent” signal is sent simultaneously to the inner ear vestibular system with a cerebral cortical instruction regarding any voluntary head and body muscles. This “pre-planning” includes a necessary response to “emergency” situations, as we have described.

## 6. Proposal for a Simultaneously Acting Interconnected Afferent/Efferent Vestibular System

**Figure 1:**
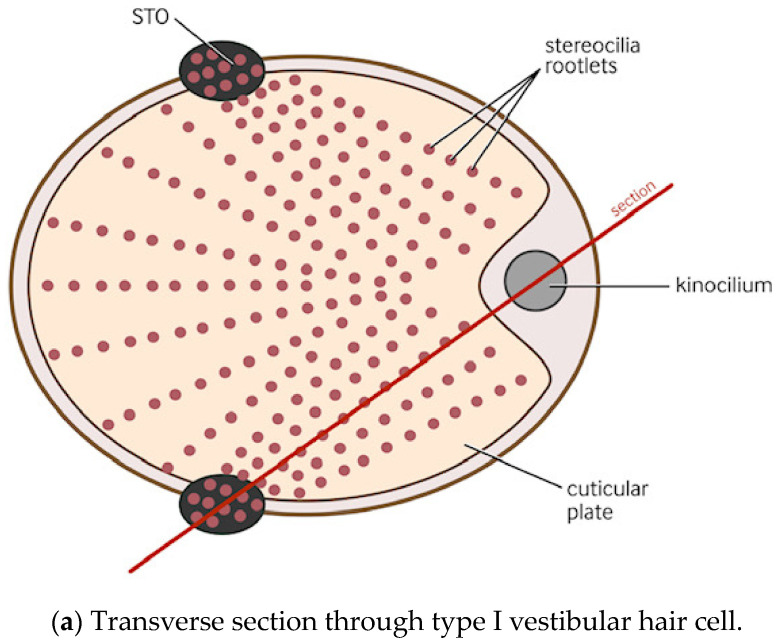

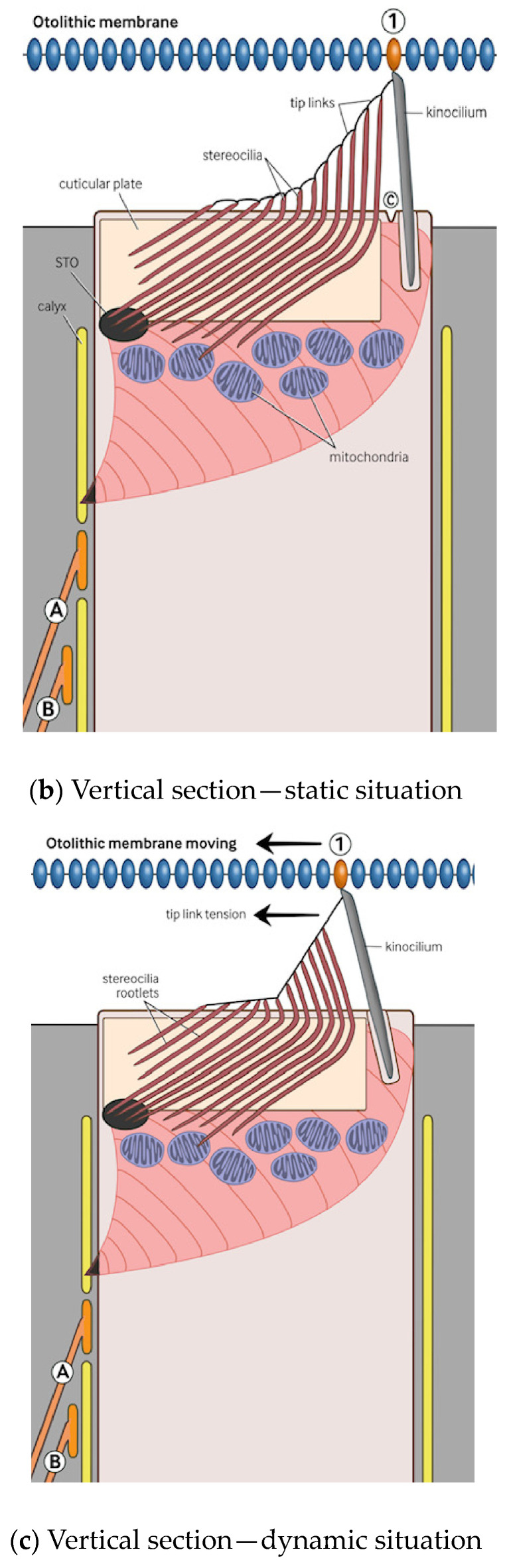
(**a**) This shows a transverse section through the cuticular plate of a type I vestibular hair cell. The cuticular plate is a bowl-shaped actin gel constructed of filaments with no directionality. The kinocilium is on the right. The dots represent stereociliary rootlets projecting down from stereocilia that have tip links to the kinocilium. The striated organelle (STO) is subcuticular. Points of extra adherence (where stereociliary rootlets preferentially tend to focus) are indicated by the black areas at the sides. (**b**) In this vertical section, the STO is in pink, separated from the cuticular plate by a dense cluster of exceptionally large mitochondria. The distal calyx of the afferent vestibular axon (shown in yellow) contacts the hair cell and reaches to just below the cuticular plate. Beneath the cuticular plate of the cell is the layer of mitochondria (for energy supply), some of which are contacted by stereociliary rootlets which have passed through the cuticular plate. The axons labelled “B” are vestibular efferents standardly placed on the outside of the calyx. The axons labelled “A” are the small number of efferent axons, which bypass the calyx and synapse directly on the apical type I hair cells. (**c**) This illustrates the “emergency” fall prevention strategy, activated by the small population of type “A” cells. The stereocilia have been displaced due to the slip. This deformed the STO. As a result, the attached kinocilium at site “1” has been displaced. This is maximal at right angles to the direction of the unexpected movement in the utricular striola. The detected direction of the rapid response will hopefully allow for a protective injury-preventing movement. This emergency response pathway provides further evidence about the extreme complexity of the vestibular system.

Human activities coincide with (and are coordinated precisely with) the amount of vestibular efferent and afferent activity at the inner ear vestibular end organ in order to orchestrate a planned movement. The aim is to have an inner ear response which is appropriately timed for the movement and of the correct magnitude. There is early recognition of any discrepancy that may have arisen due to an external influence, and corrective action is instituted if necessary. When an individual decides to make a certain movement, efferent instruction from the cerebral cortex travels to muscles in appropriate parts of the body. This coincides with planned vestibular efferent instruction to the saccular and utricular hair cells, and their stereo/kinociliary hair cell complexes are stimulated to position themselves simultaneously with the expected and probable otoconial inertial change which, based on past experience, will probably take place. The aim is to have the polarizing of vestibular hair cells and afferent axonal firing rate unchanged due to the movement. There are circumstances under which the vestibular afferent firing rate needs to be modulated. During an activity like running or walking, foot impact will vary depending on the factors such as the compliant nature of the surface or the type of footwear worn. Should the inertial movement of the otoconial membrane differ from the expected and planned movement due to an unexpected component (such as soft ground instead of a hard surface, as occurred in our personal example, or being bumped unexpectedly), the error results in a discrepancy between the expected and predicted otoconial positions associated with the movement. As a result of this, the tip of the kinocilium of the hair cell is torqued by the otoconia. This causes a change in position of the stereociliary/kinociliary complex, which generates type I vestibular hair cell membrane polarization change. Figure 1 outlines the ultrastructure of the type I hair cells. In Figure 1a, a transverse section through a type I hair cell is illustrated. Figure 1b,c illustrates a vertical section of an unperturbed (i.e., static) hair cell and an active hair cell, showing displacement of the stereocilia. This displacement results in alteration in the afferent vestibular axonal firing rate. It was shown some years ago that when the stereocilia move toward the kinocilium of the vestibular hair cells, they become less polarized, and when they move away from the kinocilium, the hair cell polarization increases [16]. In the same paper, these investigators were also the first to describe stereociliary rootlets piercing the cuticular contractile plate at the apex of the vestibular hair cells, below which they were noted to be in close proximity to numerous large mitochondria.

The “non-auditory labyrinth” with the presence of “separate sensory receptors” and the ability to detect linear acceleration was detailed some 60 years ago by Money [17]. Early anatomical understanding of the macular structure of the inner ear was limited. Because astronauts vomited profusely in space and were unsteady on return to earth, a major expenditure was provided to drive a “scientific endeavor” during the American space program in the 1960s. Studies by Spoendlen [18,19] and Lindeman [20,21], among others, discovered anatomical structural features of the macular systems. Carlström and Engström drew attention to the structure of the otolithic membrane [22]. Lim outlined that the density of calcium carbonate is nearly three-times that of the surrounding milieu [23]. In the 1980’s Anniko, using X-ray crystallography, described the mineral content of the otoconial membrane in animals and in humans [24,25].

Details of the protein lattice, which makes up a substantial component of the otolithic membrane, were later described in detail by Lundberg and others [26,27,28,29,30]. (The presence of such a substantial protein component of lower specific gravity than calcium carbonate means that, although the otolithic lattice as a whole weighs more than the surrounding milieu, the lattice itself is not three-times as dense.) However, the inertial principles we discuss here are still applicable.

The older literature outlined that click stimuli could evoke cortical responses in man [31], and that these responses were generated by the audiovestibular system [32]. However, our precise understanding of macular function was hampered, until relatively recently, by an absence of the ability to investigate these structures effectively. Our recent ability to record these responses clinically (vestibular evoked myogenic potentials or “VEMPs”) [33] and their development as a clinical tool [34] makes measurement of function of the maculae of the saccule and utricle possible in patients. These assessments are very useful, as permanent VEMP abnormalities correlate clinically with chronic balance difficulties, ongoing vegetative symptoms, and, often, substantial incapacity [35,36].

Now that testing is available, a more precise and complete understanding of exactly how the maculae work in humans needs to be developed in order to improve and optimize treatment outcomes for patients with vestibular disease. The anatomical and physiological complexity of the maculae is extreme, and because of this Young has stated “…the nerve from the macula could be regarded as a complex parallel information channel whose signal depends on the consent of many sensory cell responses to indicate movement of the head and direction of the apparent vertical” [37]. This was confirmed anatomically some 30 years later by the anatomist Muriel Ross [38].

## 7. The Vestibular Efferent System and Its Association with the Striated Organelle

The vestibular efferent system makes up about one third of the vestibular axons to the maculae [39] in the human but until recently its function was not understood [40]. It must have a significant function, as it forms such a large portion of the nerve. Recent publications by Matthews [41], Poppi [39], and Schneider [42] have suggested several functions with extremely complex biochemical signalling, but the actual role of the vestibular efferent system remains unclear. The system modifies vestibular hair cell response predictably so that only unexpected motion is detected and responded to, which is then referred centrally by afferent vestibular neurons. 

In 1963, a “Friedman body” or “striated organelle” (STO) was described [43] near the apex of the vestibular hair cells during surgical removal of the inner ear for Ménière’s disease. At first, the STO was considered to be a possible feature of that disorder. However, when an approach to the internal auditory canal through the labyrinth of the inner ear was devised [44], it was recognized to be present in the tissue of the normal inner ear [45,46]. Interestingly, the second authors on these two latter papers are two of the most prominent otolaryngologists of their day: Sir Terence Cawthorne (who detailed Cawthorne–Cooksey vestibular rehabilitation exercises) [47], and William House, who, with William Hitselburger, devised both the middle fossa and the translabyrinthine approach for acoustic neuroma removal, and also developed and popularized cochlear implantation technology worldwide after its initial description by Djourno and Eyries in France [48].

The STO is found in all labyrinthine vestibular hair cells in advanced animals and in cochlear inner hair cells. Early recognition of the presence of the STO as a consistent labyrinthine structure might have led to its inclusion in the theories of function of labyrinthine cells, but, despite its consistent presence in advanced animals, its significance has not been appreciated, understood, or taken into account, even subsequent to a recent detailed serial section electron microscopic structural description by Vranceneau et al. in 2012 [49], and this structure surprisingly continues to receive little attention. It is suggested that the STO might serve two functions: to maintain hair-cell shape and to alter transduction by changing the geometry and mechanical properties of hair bundles [49].

A detailed description of the STO is necessary. As indicated in Figure 1a, it is a structure located in the subcuticular region of vestibular hair cells, consisting of alternating thick and thin bands of contractile protein [43,45,46,49,50]. It is present in both type I and type II vestibular hair cells and inner cochlear hair cells. The STO is particularly well-developed in type I vestibular hair cells and is shaped like an inverted open cone made up of contractile protein. The apex of the cone pierces the type I vestibular hair cell membrane on the opposite side of the cell to the kinocilium and directly contacts the calyx surrounding the cell (illustrated in Figure 1b,c). The base of the inverted cone of the STO contacts the cell membrane along the circumference of the cuticular plate, particularly at two points at opposite sides of the hair cell, with a third point to which it attaches in the cell membrane near the base of the kinocilium. It has been speculated that in conjunction with the stereocilia, the STO may have a role as a mechanotransducer [48]. Its circumferential adjacency to the cell membrane at the cuticular plate suggests that it may help in the formation of the constricted neck (which is characteristic of type I vestibular hair cells). Inside its expanding core are many very large mitochondria. The STO is separated from the stiff layer of the cuticular plate (which contains actinic and other contractile proteins) by this layer of mitochondria. Using electron microscopic (EM) tomography in type I vestibular hair cells, it has been shown, in three dimensional reconstructions, to be connected to at least some stereociliary rootlets which pierce the cuticular plate. The contact with the rootlets indicates that the STO regulates stereociliary hair bundle activity, and the large mitochondria surrounding it indicate that it has a very high energy requirement. Although studies have outlined the critical importance of hair bundles and mechanotransduction to vestibular function [51,52], the significance of the STO has not been emphasized or taken into account in theories of vestibular function.

The red line labeled “section” in Figure 1a indicates a vertical section taken through the hair cell, which is illustrated in the Figure 1b,c.

The kinocilium is attached to the otolithic membrane at site “1”. It leans slightly towards the centre of the cell and can flex in either direction. Its contact with the rootlets means that this kinociliary/stereociliary complex can respond to a sudden unexpected otolithic displacement.

It is notable that after intratympanic administration of gentamicin, a high concentration of gentamicin was confirmed near the apex of type I vestibular hair cells (where these large mitochondria are located) [53]. The mechanism of aminoglycoside action is to bind to the 30S subunit of the ribosome. Given our present understanding of aminoglycoside therapy and ototoxicity, one would expect that structures with such a high metabolic rate that are so closely allied anatomically to the mitochondria would be preferentially affected by a vestibulotoxic agent [54], with disruption of mitochondrial ribosomal activity and resultant cell death [55]. The acute loss of semicircular canal function resulting from gentamicin vestibulotoxicity results in oscillopsia (as a result of total bilateral loss of VOR function).

In the tubular type II vestibular hair cells, the STO is a much smaller structure and appears to be free-floating in the cell apex below the cuticular plate [49]. Our understanding of them is poor, but recent research has shown that there is some biochemical mediation of calcium in synapses. 

As discussed previously, our “efferent/afferent” proposal is that when a movement is to be initiated, the cerebral cortex sends an instruction through brainstem/cerebellar pathways about this intended movement of the head, the body, or both, and a series of muscle responses is set up to have this occur. Along with the intended motor activity, the vestibular efferent nerves simultaneously inform the vestibular macular hair cells via brainstem pathways from the cerebral cortex through the vestibular efferent system (VES) that this event is about to happen; i.e., that inertial movement of the otoconial membrane is about to occur. The VES causes a polarization change in the type I vestibular hair cells, which causes the STO to position the stereo/kinociliary complex for the predicted otoconial move. This repositioning coincides closely in time with the movement of the otoconia, which move simultaneously, so there is little change in vestibular hair cell polarization due to this apical hair cell activity. Given the importance of maximizing the speed of this response, it is necessary that the VES be in close proximity to the STO.

This rapid response system also requires some flexibility so that it can deal with subtle changes in normal ambulation behaviour, such as encountering a different walking surface (e.g., a rubberized track) or even the influence of footwear types (e.g., gel inserts). In brief, some flexibility of afferent input must be accommodated.

## 8. Macula Hair Cell Innervation

In order to outline how the proposed system works, it is necessary to describe the anatomy of macula hair cell innervation. Type I and type II vestibular hair cells are distributed throughout the macula of the saccule and utricle [4]. While type I vestibular hair cells are predominantly intrastriolar, type II cells are largely extrastriolar. The percentages of type I vestibular hair cells which are extrastriolar and of type II vestibular hair cells which are intrastriolar are based on species-specific balance requirements.

As summarized by many authors (e.g., [5,39]), primary vestibular afferents form large cup-shaped calyx terminals that envelope the basolateral surfaces of the hair cells. Unlike auditory afferents, the vestibular calyx is postsynaptic.

The peripheral endings of the afferent vestibular neurons consist of three types:

Type I hair cells are almost completely enveloped by this cuplike terminal. Each ending can surround more than one hair cell and there are multiple intracellular hair cell synaptic ribbons distributed directly adjacent to the calyx. In some instances an efferent bouton remains in direct contact with the type one hair cell, so the calyx surrounds, not only the type one hair cell, but also covers the remaining efferent bouton and surrounds its efferent axon. The efferent ending and distal axon are represented in Figure 1b,c as neurone A.

Type II hair cells have a more cylindrical or columnar morphology and are contacted by typical bouton-like afferent terminals. They have a single synaptic ribbon on the hair cell below each bouton. Boutons also supply more than one type II vestibular hair cell.

A peripheral vestibular afferent neurone is formed when calyceal endings from several type I vestibular hair cells join together. In addition, a similar process occurs with type II vestibular hair cells, and sometimes afferent endings from type I and type II vestibular hair cells fuse together to make up the distal end of the peripheral axon [19].

Detailed cell counts in several species of animals indicate that there are many more axons than hair cells. Dimiccoli et al. [56] indicated the complexity of this process, suggesting that only two or three hair cells are connected to an afferent neuron. However, the earlier work of Gacek and Lyon [57] and Gacek [58] outlined that even five or six hair cells may be connected to each neuron, making the afferent interlinking process even more complex to analyse. Vestibular afferent neuron interlinking allows each neuron to respond to the activity of multiple hair cells [56], perhaps with slightly different orientation. This results in peripheral vestibular hair cell activity undergoing neural integration of information and preliminary analysis before reaching the vestibular nuclei in the brainstem; a similar process also occurs in the eye [59].

Our ability to use VEMPS in the clinical setting allows us to measure saccular and utricular responses to sound and vibration, and this makes it possible and worthwhile to theorize and break down the “integrated system” (proposed physiologically by Young [37] and anatomically by Ross [38]) into component parts to produce a precise framework of function. With this ability, clinical treatments will hopefully be devised. As a result, damage to these structures could be controlled and repaired, which will assist in dealing with advancing vestibular disease.

Further studies have helped us understand the biochemical and physiological activity taking place in the vestibular maculae. Type I vestibular hair cells are surrounded by the distal calyx of the first-order afferent neuron. Analysis of the zones of calyces around individual type I vestibular hair cells has shown four domains with different biochemical activity [58]. Specific details of the biochemistry are beyond the scope of this article but domain 2, which is located at the apex of the calyx closest to the cuticular plate, the cell apex, and the otoconial membrane have been shown to have less substantial biochemical activity, if any at all, although it is possible that new biochemical processes may have yet be discovered in this location [59]. Importantly, this is the location from which the apex of the STO originates. It has been suggested that absence of biochemical activity at this site may be explained by the fact that this is a region where calyx activity directly influences the type I vestibular hair cell [59].

The apex of the STO cone in domain 2 extends through the vestibular type I hair cell membrane directly into the calyx membrane [59]. This is shown in Figure 1b. The action and activity of the efferent bouton vestibular endings have also been shown to occur on the calyx [49] but, as discussed above, a small number of VE endings insert directly into the type I cell vestibular hair cell in several animal species [57,60,61]. As illustrated in Figure 1c, our proposal is that, the STO generates kinociliary/stereociliary push/pull contractile protein activity (contraction or relaxation), which coincides precisely with the expected and predicted otoconial momentum shift due to the preplanned physical movement of the head, the body or both, by using this direct calyx contact.

**Figure 2:**
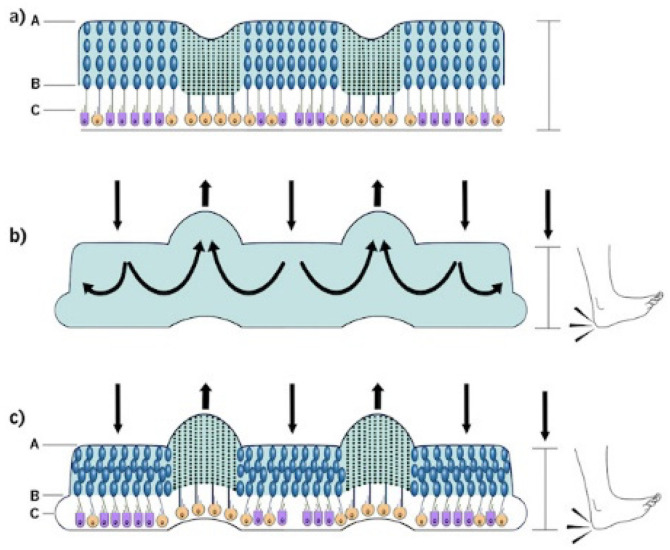
Effect of foot impact.

In activities such as walking or running, when a heel strike impact occurs, (Figure 2) a cerebrocortical vestibular movement command is given. This results in a contraction or relaxation of the STO, resulting in a change in kinociliary position due to the STO’s contractile protein activity. The resultant movement pulls (or relaxes) the cell membrane at that location (i.e., a “dimpling” at the base of the kinocilium). This response is maximal at the location in the macula most near at right angles to the direction of the maximal movement. As a result, the kinocilium moves precisely with the otolithic displacement caused by the movement, and no vestibular afferent impulse rate change is needed or generated. This is a high-energy-demand activity, which explains the presence of, and need for, the large mitochondria to supply the STO and cuticular plate contractile proteins, which drive the stereociliary/kinociliary complex.

This action struck the authors as being analogous to the role of tropomyosin, a “coiled coil” structured protein that is crucial in the regulation of actin–myosin contraction in muscle. It has been detailed that sterociliary rootlets are actin-based structures, that they are essential for the structural stability of the stereocilia in order to tolerate innumerable deflections over their lifetimes, and that they utilize actin and its associated proteins to carry out the function of mechanotransduction in this unique manner [62]. Studies have localized tropomyosin within the stereociliary rootlets and suggested that it may control the access of actin-binding proteins to the rootlet structural filaments [63], but recent work has also localized several other contractile proteins to the rootlets directly [62]. There are two areas where there is greater attachment of the STO and the stereociliary rootlets (shown in Figure 1a). 

Mechanotransduction is the function of the STO and the stereocilia. At these critical sites where these push/pull events are coordinated in the type I vestibular hair cells, motility of the kinocilium is also altered as the mobility of the cuticular plate beside the kinocilium is altered by contraction or relaxation of the contractile protein of the striated organelle. The stereocilia are tensed or relaxed by the contractile proteins at their base in the cuticular plate in a push/pull effect and the reverse process also occurs. In other words, as the kinocilium tenses, the stereocilia relax. This is closely coordinated with the predicted and expected otoconial movement due to vestibular efferent instruction related to any head movement. In brief, the type I cell has an intracellular contractile/relaxation process resulting from direct vestibular efferent stimulation. During a normal movement, the movement of the kinocilium coincides with the expected otoconial membrane movement and no change in the polarization of the hair cell membrane occurs. If the otoconial membrane position is different to what is expected and predicted, (due to an unexpected and excessive depolarization, e.g., our “soft ground” example) this causes a polarization change in the cell wall, and the result is a change in the firing rate of the vestibular afferent neuron. The change in polarity of the cell wall of the vestibular hair cell at this site results in alteration in the length of the contractile protein of the STO. These abilities are learned early in life. This is followed by “training” to coordinate the otoconia and hair cell activity as an individual acquires the complex skills of bipedal gait.

The ultrastructure of the type I hair cell enhances the speed and precision of its response. The STO response to efferent vestibular instructions via the push/pull effect is to coordinate precisely with otoconial movement during velocity change. The fact that the proximal end of the type I vestibular hair cell striated organelle bundle pierces the cell wall of the hair cell, and even the cell membrane of the calyx wall, certainly maximizes the speed and precision of its effect, as this allows for a more precisely timed and rapid connection than would be afforded by a standard chemical synapse [49].

## 9. Vestibular Efferent “Bypass” Neurons

A further layer of anatomical complexity that may help a biped initiate the previously described “slip and grip” strategies has recently been described. There are a small number of vestibular efferent axons that bypass the calyx, the significance of which appears to have been ignored in many papers [40,57,60,61]. We suggest that this is part of the “emergency response” system. In our “slip and grip” scenario, this small number (5 to 10%) of efferent axons which bypass the calyx and synapse directly on the apical type I vestibular hair cells (labeled neuron “A” in Figure 1b,c) facilitate the transmission of a rapid cerebral cortical signal as soon as the initial slip is detected. This is one of the several activities that this poorly understood vestibular efferent system undertakes, as mentioned earlier. In this situation, the STO is required to act in both directions. In the first step of this response, the cerebral cortex estimates the probable otoconial movement, its direction, and its extent caused by the slip. This information is sent as urgently and as rapidly as possible to the stereociliary/kinociliary complex via the population of efferents that bypass the calyx and contact the hair cell directly. This is followed by rapid feedback of any error in this otoconial estimate, as the otoconia tweak the kinocilium of the stereociliary/kinociliary complex. This emergency stimulus is fed back through the STO directly via its apex to the calyx. As discussed, this response is maximal at right angles to the maximally detected otoconial movement. At the same time, the standardly placed vestibular efferents, on the outside of the calyx (labeled neuron “B” in Figure 1b,c), facilitate a burst of efferent stimuli, causing the irregular-firing vestibular afferents to increase the dynamic range of response of the maximally stimulated macular region at right angles to the slip. This is the last-ditch emergency attempt to either prevent a fall or to maximize protective body movement if a fall becomes inevitable.

The type II vestibular hair cells serve a slightly different purpose, as they are more “distortion sensitive”. In these cells, vestibular efferent axons directly contact the vestibular hair cell membrane and the afferent first-order neuron, causing a change in polarity with resultant kinociliary tensing or relaxation via the striated organelle to maintain cell shape during otoconial movement. The biochemical complexity of calcium metabolism in type II hair cells is poorly understood, but the shape and extent of the distortion as well as its severity indicate an awareness of body angle with respect to the direction of gravity and velocity [49]. There is no stereociliary rootlet attachment through the cuticular plate to coordinate the kinocilium and stereocilia in type II vestibular hair cells. The amount of distortion the striated organelle incurs (as a result of contractile protein activity) indicates the effect of the orientation change due to gravity alteration during a movement. A change in the firing rate of the type II vestibular hair cell may be required if the distortion from a movement and its changed orientation to gravity is not predicted correctly for the planned movement due to an external factor, such as ground softness or a change in shoe wear, as discussed above.

## 10. Significance of the Location of the Line of Polarity Reversal in the Maculae

Ultrastructural differences between type I and type II vestibular hair cells have been described by Spoendlin [18] and Lindeman [20,21], who detailed the reversal of direction of polarity in the striola of the utricle and saccule. Both type I and type II hair cells have kinocilia facing each other in the striola of the utricle but facing away from each other in the striola of the saccule, although, depending on the species, the numbers and types of hair cells located in these areas vary [64]. As detailed in Figure 2, it can be seen that for any predicted movement of the head, there are kinociliary and stereociliary movements in opposite directions on either side of the line of polarity reversal (LPR).

Figure 2 shows an anteroposterior transverse section through the macula of the utricle. The large extrastriolar otoconial lattice is represented by large blue dots (between “A” and “B”), and the striolar otoconial lattice is shown with small blue dots. Because the utricle is U-shaped, with the apex of the “U” facing anterolaterally, the striola is shown to be transected twice. The line of polarity reversal (LPR) is shown at the lateral margin of the striola, where the kinocilia of the vestibular hair cells face each other. The space below the otoconial lattice (between B and C) just above the surface of the type I and type II hair cells (layer C) is filled with gel. The stereocilia and kinocilia of the sensory vestibular hair cells project into this gel.

When a heel strike (foot impact) occurs, a cerebrocortical vestibular movement command is given, resulting in a contraction or relaxation of the STO.

The resultant movement in the contractile protein of the STO pulls (or relaxes) the cell membrane at that location (i.e., a “dimpling” at the base of the kinocilium). This response is maximal at the location in the macula most near to right angles to the direction of the maximal movement.

More recent work [52] has demonstrated that in the macula of the utricle, the LPR of vestibular hair cells is located at the outer peripheral edge of the U-shaped striola, rather than being under the middle of the striola as previously thought. Because the LPR is situated at the site of adjacency of large and small otoconia in the utricle, movement of gel due to velocity change is maximized. In the saccule, the LPR is under the middle of the striola [52,65,66].

**Figure 3:**
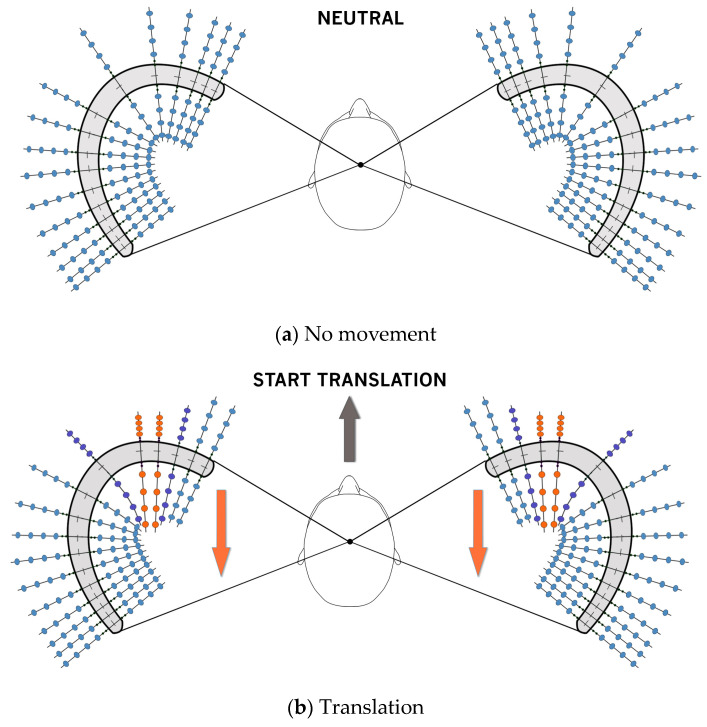

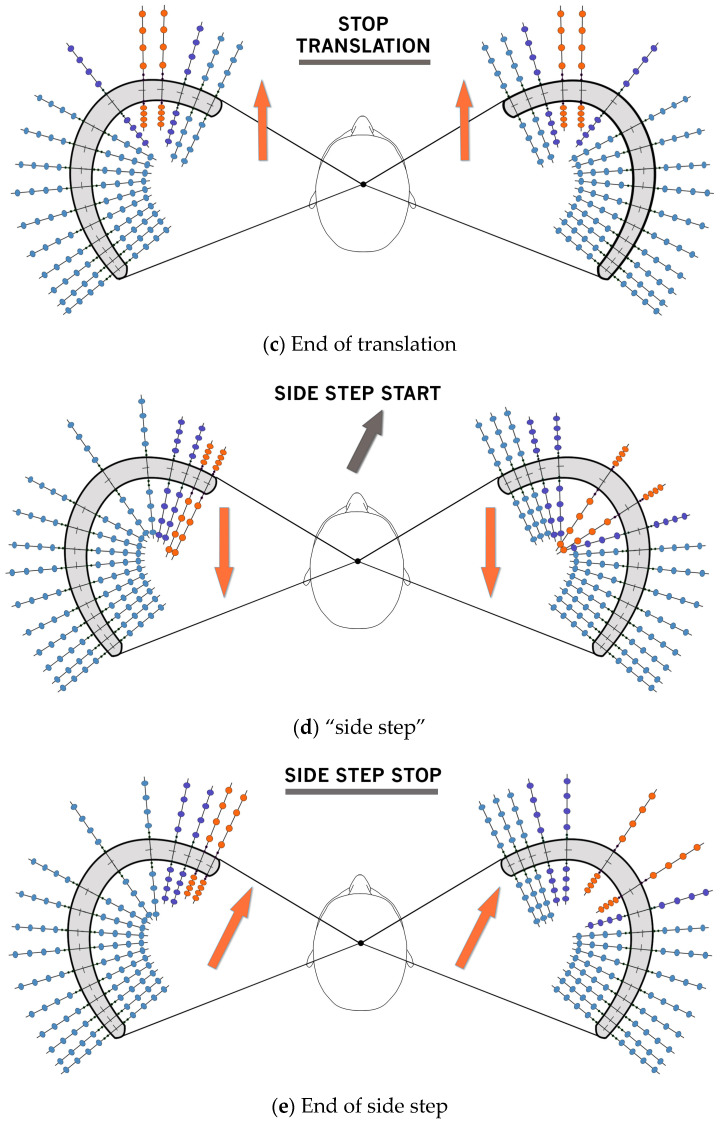

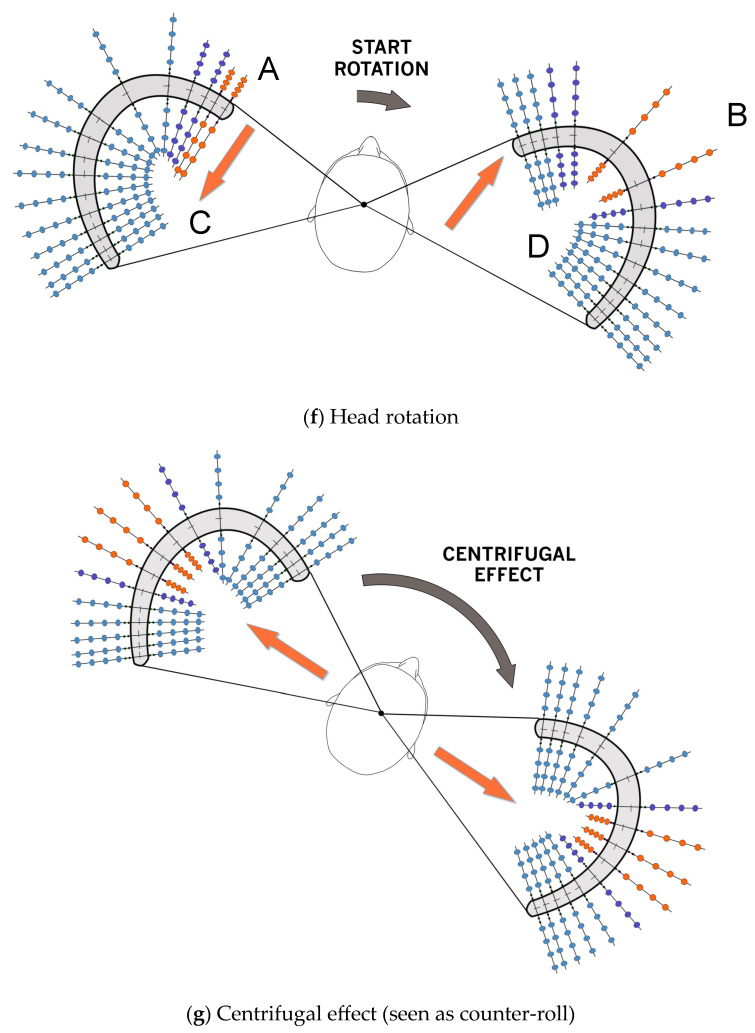

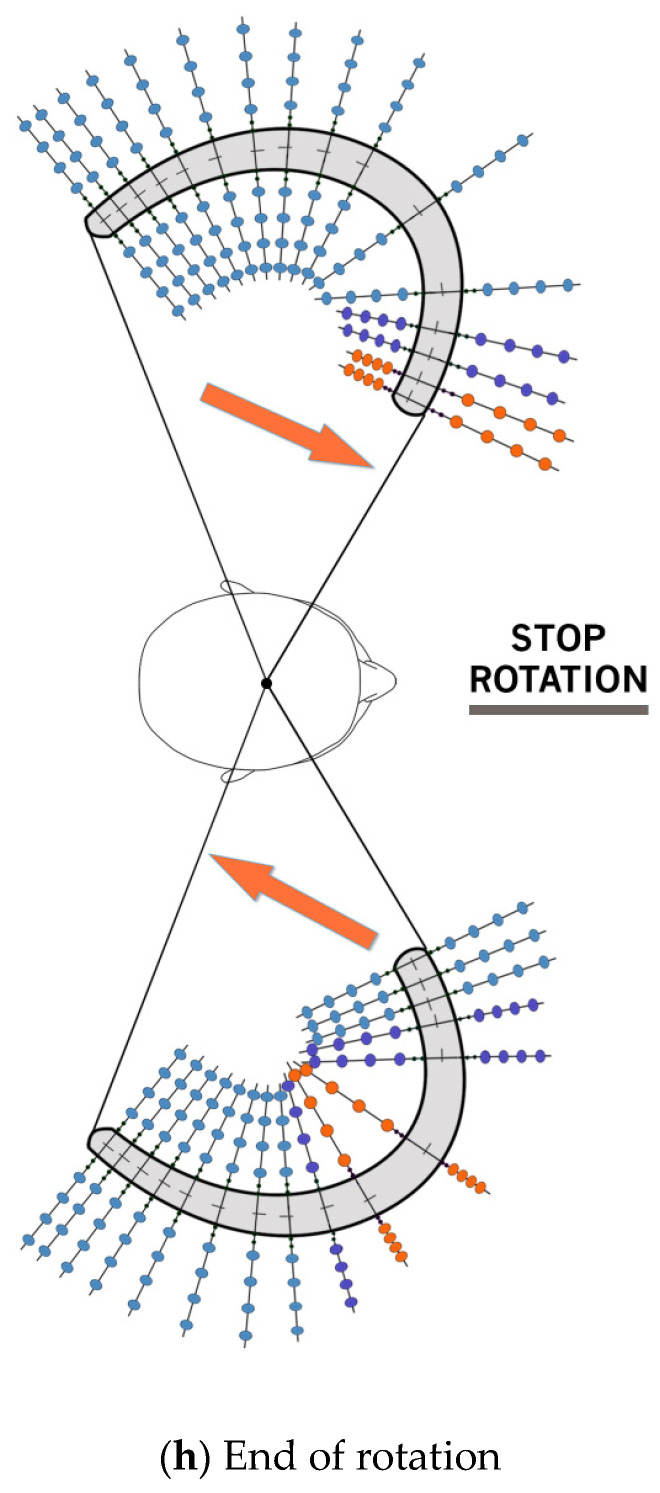
Utricular orientation.

It is essential that the orientation of the utricle is accurate so that theories for how it functions are based on an anatomically correct model. Such a model is illustrated in Figure 3a. Lindeman [20,21] and others have documented that the differences in the shape of the macula and the number of type I and type II vestibular hair cells differ between species, dependent on their particular evolved needs. The exact shape of the mouse utricle differs slightly in shape and orientation [52] from that of the human [67], given that a quadrupedal animal has different head and body movement requirements. In all animals, it is logical to assume that the maximal inertial stimulation during any head movement is at right angles to the striola otoconia LPR [68].

This series of diagrams of a human utricle represents a view looking down on the utricular maculae of both sides. This shows the “U”-shaped utricles bilaterally to the head in a static position. Note that the trailing posterior arm of the “U” is a “lazy U” with an elongated arm. It only completes a 160° turn, as opposed to a full 180° horseshoe (as is usually drawn in medical textbooks).

The large extrastriolar lateral and medial otolithic lattice columns are shown in blue. The striolar area is in grey. The large dodecahedral otoliths are approximately 30 nm in size, while in the striola they are only 2 to 4 nm. For diagrammatic purposes, the extrastriolar small otoliths are shown with a gap between the anterior and the posterior arms. This gap is filled with the otoconial lattice in the living situation.

During a forward translation of the head, the region where maximal inertial movement occurs is shown in red. Note that the otolithic lattice is crushed together compared to that of the surrounding medium due to its relative mass. As the translation is equal in both utricles, responses on both sides are the same. The most active otoliths (in red) behind the striolar area are maximally strung out. The adjacent area (dark purple) shows a relatively increased, but not maximal, movement.

When this translation stops, the results are the exact opposite of the effect illustrated in Figure 2.

When an angled forward side step is taken (i.e., “off to the side”), movement of the otoconia is at right angles to the direction of movement and results in them being ”bunched up” or “strung out”, (i.e., similar to the forward movement shown in Figure 2). The movement of the lattice differs on the two sides but continues to be maximal at right angles to the direction of the side step.

At cessation of the side step, (Figure 3e) there is an exact reversal of the movement seen in Figure 3d.

As the head turns to the right, the initial inertial delay results in the otoconial lattice moving in exactly the opposite direction on each side of the head. During a head turn to the right, the otoconia on the leading anterior (A) and trailing posterior arm (D) (shown in red) bunch up, while the leading posterior (B) and trailing anterior (C) otoliths string out.

Otoconia at the centre of the laterally orientated “U” (shown in red) move peripherally on both sides due to the centrifugal effect of the momentum of the large otoconia. This results in bunching up on the inside of the “U” and stretching out on the outside.

The otoconia undergo the reverse process to the initial movement shown in (f) and return to the neutral position shown in (a). This causes the red otoconia to bunch and stretch appropriately.

Figure 3b,c shows a simple forward translation while looking straight ahead. In this situation, there is only one site in each utricle that is maximally stimulated at right angles to the LPR of the striolar direction of movement. A more complex 30° lateral movement while continuing to look forward (i.e., a sidestep) is detailed in Figure 3d,e. Notice that this results in maximal stimulation at right angles to the movement on the side opposite to the direction of the movement, but at only one site on each side.

Figure 3f–h shows the response of the utricle between the start (i.e., acceleration phase) and stop (i.e., deceleration phase) of a head rotation, as opposed to a translation. Figure 3g illustrates the centrifugal movement of the otoconia, which clinically elicits eye counter-rolling that is demonstrated during eccentric chair rotation [69]. Depending on the change in head position (such as turning the head to look sideways as one starts running), there is a change in the position of maximal stimulation at right angles to the vestibular hair cells of the striola (due to the complex movement that has taken place).

Due to efference copy experience, this results in the ability to differentiate head movement (e.g., turning) from independent body movement (e.g., running) that occur at the same time.

**Figure 4:**
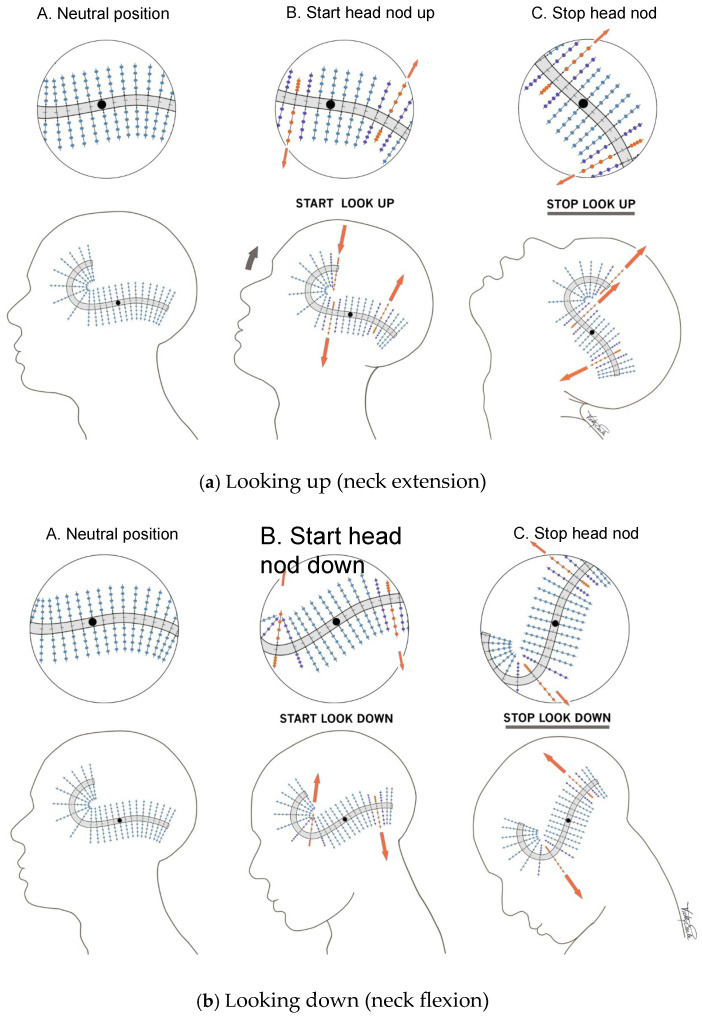
Angular movement.

Figure 4a,b shows a diagram of neck extension and flexion (i.e., an up-and-down head movement, or “head nod”). During neck extension, the saccule has up to three points where stimulation occurs at right angles to the LPR of the striola. If there is an anteroposterior change in velocity at the time of head movement (for example starting to run while simultaneously hyperextending the neck to look upward,) this will result in a vector for maximal stimulation at right angles to the LPR, so the point of maximal stimulation at these three points moves. Although we think of these movements as smooth, it is again not velocity but acceleration or deceleration (i.e., velocity change) that is detected. This is because head acceleration (at the beginning of a head movement) and deceleration (at the end) are brief and velocity is constant throughout the central period of the movement. Depending on the angle of the head nod, maximal stimulation at right angles to the LPR may occur at either two sites (during neck flexion) or three sites (during neck extension).

Given the ultrastructure of vestibular hair cells and using our proposed theory about how the maculae cope with a given response, we can now analyse in detail the mechanisms which deal with our brief “foot slip and grip” event (something that humans are familiar with). If the normal response is not sufficient, the result may be a crisis, perhaps with a fall as a consequence. There is little time for a cerebrocortical response via the vestibular efferent input, but the few “direct contact” efferent axons discussed previously are fast and can approximately position the most active kinociliary/stereociliary complex that is at right angles to the slip. They accomplish this by acting directly on the type I hair cell and thus the STO. They then receive otoconial input as the slip (which occurs at right angles to the maximally detected otoconial movement) proceeds. This input may indicate that a slight adjustment of response direction is necessary. Because the apex of the STO is embedded in the calyceal wall, the speed of afferent information transfer is maximized. In this emergency situation, the STO acts in both directions: first, with an efferent process to recognize and approximate the direction and extent of the otoconial movement that has been caused by the slip; secondly, with a rapid signal to feed back any error in the original estimate. At the same time, in an attempt to prevent fall or maximize protective body movement if a fall becomes inevitable, the standardly placed vestibular efferents on the outside of the calyx facilitate a burst of afferent information through the irregular firing vestibular afferents from the maximally stimulated utricular region at right angles to the slip [42].

This illustrates the effect of head nodding on the macula of the left saccule. The black dot represents the fulcrum around which extension or flexion occurs. The site of maximal large extrastriolar otoconial lattice displacement is shown as columns. The area with maximal effect is at right angles to the direction of movement and is shown in red. Due to the “lazy S” structure of the saccule, there are three places at which the otoconia are maximally stimulated at right angles to the movement. During extension, the two sites anterior to the fulcrum (shown by the arrows) move in the same direction, while the posterior end behind the fulcrum moves in the opposite direction. In some movements, (e.g., if the angle of the head nod changes), there may be only two active right angle sites, in which case they are in opposite directions.

The area with maximal effect is again at right angles to the direction of movement and is shown in red. The otoconia are again maximally stimulated at right angles to the movement. Once again, the posterior end behind the fulcrum moves in the opposite direction to head movement and there are only two active right angle sites, moving in opposite directions.

## 11. The Complexity of the Vestibular Efferent System Makes It Difficult to Assign Clearcut Functions

The detailed elegant experiments undertaken by Jamali et al. [70] and Sadeghi et al. [40] on chinchillas used “non-emergency” repetitive predictable controlled movements. Cullen and Wei have very recently shown little difference in afferent vestibular neuronal responses between active and passive head movements [6]. In other words, the vestibular efferent system appears to not be involved in these activities. This includes running, where these authors showed increased afferent firing by the irregular afferents. However, a careful statistical calculation resulted in them discounting this observation. They emphasized that “the function of the in everyday mammalian vestibular efferent system life remains poorly understood”, and that “it appears as if the vestibular efferent system does not play a role in short-term modulation of afferent coding, but instead plays a role in modifying sensory encoding over a longer time course”. Caution must be exercised in trying to correlate the absence of vestibular afferent consequences of rhythmic movement in the chinchilla and rhesus macaque with the jerk effect in the human (i.e., when the foot hits the ground) on the otoconia of the maculae of the saccule and utricle, as this is more of a vertical energy transfer. The mechanical effect on the impact in this situation is substantial, and modification of the impact cancellation system is probably necessary [6].

## 12. Emergency Fall Prevention Strategies: The Vestibular Efferent System and Striated Organelle

We propose that slip and fall management is a main function of the vestibular efferent system in humans. It maximizes efficiency of this strategy by reducing afferent and efferent feedback time to the cerebral cortex for central decision making. As already stated, this is accomplished through a two-way push/pull effect of the STO on the stereociliary/kinociliary complex. Experiments to study this will be very challenging as the process is so brief, and once the animal is expecting the movement, it ceases to be a threat and rapidly becomes part of routine body movement responses, which are not associated with afferent neuronal firing rate change. In addition, assessment of “fight or flight” reflexes, where rapid unexpected movements are required, would require repeatedly terrifying an animal, which would be difficult to have approved by a present-day animal ethics committee.

How is this manifested in the clinical setting? Patients referred to tertiary care vestibular clinics sometimes describe non-emergency sensations, such as “having to catch up with a head turn” or a feeling that they are still turning, after movement is completed. In the authors’ opinion, these sensations represent mistimed coordination of otoconial and kinociliary movements due to disease of the maculae, manifesting as these clinical complaints.

As outlined in our “slip-and-grip” scenario:

Reliable proprioceptive information can be suddenly lost,

The visual information is confused by the rapid head movement,

The “go to” system for stability is the inner ear balance system, along with rapid cerebral cortical decision making.

The result of this will be a (hopefully successful) physical response, so that a fall (with a potential head strike or other injury) can be avoided. Using vestibular information, a rapid step forward or to the side, or putting an elbow out when falling backwards, can reduce or prevent significant injury. These responses are usually carried out successfully, as most adults are aware of the consequences which they “learned the hard way” during learning to balance, walk, and run in childhood.

## 13. Trauma and Otoconial Disruption

The concept of cupulolithiasis was first put forward by Schuknecht [71], and confirmation of its occurrence was made by observation of otolithic debris in the posterior semicircular canal by Parnes and McClure in patients with benign paroxysmal positional vertigo (BPPV) [72]. Cupulolithiasis is very common following trauma [73], as evidenced by the high level of otolithic pathology (VEMP abnormalities) that is seen post-trauma [74]. Clearly, otoconial detachment occurs when there is an acceleration/deceleration injury. The stereociliary/kinociliary complexes are attached at their distal end to the undersurface of the otolithic membrane. There is compelling evidence that free-floating otoconia displaced from the otolithic membrane in the utricle underlie most cases of BPPV [72].

The hair cell bundle is detached with an acute acceleration/deceleration injury. This most likely involves the large peripheral otoconia, as they are heavier and more likely to be torn free and float into the posterior semicircular canal to cause BPPV. This detachment most markedly occurs where the acceleration/deceleration force is most nearly at right angles to the otoconial membrane, resulting in specific areas of damage. Severe injury may result in extreme damage to the specific hair cells and resulting demise. This may explain why some patients incur permanent malfunction, including persistent imbalance and nausea as well as visually induced dizziness [75] (a symptom set now referred to as Persistent Postural Perceptual Vertigo, or PPPD) [76]. Another possibility of persistent symptoms is that the production of the otoconial protein matrix by supporting cells is disrupted due to injury, so that a normal otoconial membrane cannot be reconstructed. Attempts at repair may explain fluctuating ongoing symptoms as is seen clinically, as the attempted repairs fail to produce normal structure and function.

## 14. Effect of Trauma on the Hair Cells

Although traumatic injuries from falls or head blows from objects can cause disturbance in balance on a long-term basis, motor vehicle accidents are an extremely frequent cause of this [74]. The velocity of vehicles during these events and the consequential decelerative force is substantially above what would be expected in a normal terrestrial environment and, as a result, there is much more frequent and severe incapacity than would occur from a more naturally occurring (non-mechanical) event.

Over the eons, vestibular hair cells have become inured to trauma from head hits due to falls and unexpected head blows. Reasonable speculation posits that when suddenly jerked severely during an accident, the kinocilium detaches at its junction with the otoconial membrane, and the jerk also detaches the link with the stereocilia. Due to detachment occurring at structural weak points, the superior surface of the hair cell is protected. This protective process is sufficient to maintain enough vestibular hair cell integrity so that repair can occur. Symptoms of incapacitating nausea and vomiting, vertigo, and imbalance that result following an injury cause a patient to rest, usually prostrate, while symptoms settle. Reattachment at the site of acute disruption at the stereociliary/kinociliary tip link and at the kinocilium to the otoconial membrane is associated with relatively rapid symptomatic improvement, and the patient’s complaints of vertigo, nausea, and imbalance needing bedrest allow the repair process to be carried out, as movement of the stereociliary/kinociliary complex is low when there is little physical activity, which allows for ease of reattachment.

The initial premise of this paper is that accurate body orientation is associated with survival. A damaged system due to disease or age, causing poor balance, results in vulnerability. This is why an inadvertent slip during normal activities is so alarming in modern times. It causes immediate focus, anxiety, and attempted correction, but also often induces a catecholamine release and a resulting thrill some of us feel during a “fight or flight” response. This may be a major reason that we enjoy risky sports, such as downhill skiing, motorcycling, galloping a horse, skateboarding, and mountain biking [77]. 

## 15. Macula Consequences of Space Flight

Reports from astronauts post-flight have reflected the effects of “returning to gravity”. Ross has described accumulation of extra otoconia in microgravity [78]. Anecdotal reports of disorientation post-flight are likely in keeping with the autonomic responses (i.e., symptoms) often generated by a change in vestibular function, and are similar to what is often heard in the clinical setting [79].

Regulation of upright stance depends on visual, vestibular, and somatosensory feedback. The response process allows for a movement to be altered more accurately at any instant during an activity in response to an external stimulus/internal prediction error, or to an external sensory variable resulting from a change in activity (and therefore changed prediction of position). This minimizes the error and perhaps prevents injury. Sudden unexpected disturbances are compensated by an early set of almost reflex-like muscle responses that, depending on the starting position, are released without feedback control. As discussed earlier, this response is reasonably, but not perfectly, accurate [40]. It is designed to detect error due to external factors such as soft ground or an inadvertent bump. The classic example of this is the tendon reflex protective stretch mechanism, as is illustrated by the “cricket match lurch” detailed previously. 

## 16. Efferent Muscle Responses to Routine Movement and during Emergency Strategies

Although proprioceptive information from the body is necessary for balance maintenance, this article, focused on the vestibular system, is not the place to have a detailed discussion about peripheral proprioceptive function. However, similar to the vestibular system, much of our proprioceptive information, (joint position sense from the capsules of joints, the Pacinian corpuscles, as well as the Golgi tendon organs (GTOs) and muscle spindle activity) is intrinsic. Muscle contraction commands are induced by a cerebral cortical decision [15]. It is probable that cerebral cortical efferent information, with respect to intended movement, is fed into limb proprioceptors to produce a prediction of movement. GTOs, muscle spindles, and Pacinian corpuscles (particularly in joint capsules but also including skin and soft tissues) play an important role in peripheral neural control of movement. Their role is to provide the central nervous system with afferent feedback about local stresses caused by forces in muscles, including adjacent muscles [80]. The role of the GTO in this planning is essential, given its ability to detect muscle forces not only in its own muscle tendon but also in adjacent muscles.

These cerebral cortical motor control instructions are coordinated precisely with the vestibular efferent process, as previously described here. This prediction must exactly match the vestibular efferent signal from the inner ear. This predicts the exact and expected amount of body movement and the new body position for any muscle group contraction.

Afferent feedback of errors in planned movements due to external forces or conditions (used clinically as the tendon reflex response) and expected body position takes place very quickly. When considered in the context of sensory information, the purpose of efferent pathways is to provide the nervous system with the ability to adjust its own view of the external environment. This is well detailed by Mathews [41]. But, how does the vestibular system help to accommodate for sudden adjustments to cope with the cricket match lurch example detailed above? (Note that this is an adjustment which must be made immediately to prevent injury.) We surmise that this is carried out by the vestibular efferent system (VES). It informs the maculae of the utricle and saccule simultaneously, with the relevant intrafusal muscle spindles being informed at the same time as the muscle is instructed to activate.

## 17. Conclusions: The Not-Yet-Fully Understood Vestibular Efferent System

The functional role of most efferent systems in the nervous system has been characterized. This efferent projection may sensitize primary afferents to detect changes in muscle length, as well as deviations from intended movement, and extend the dynamic range of spindle responses. Auditory efferents (the olivocochlear system) have been implicated in noise protection, sound localization, and the ability to discriminate signal from background and bodily noise. However, despite over sixty years of research, we are yet to ascribe a conclusive functional role to the VES. In the somatosensory system, feedback from muscle spindles can be modulated by gamma (fusimotor) motor neurons. While there is a consensus regarding vestibular efferent morphology, location, and action on peripheral vestibular hair cells and primary afferents, a distinct functional role in motor and vestibular coordination has not yet been ascribed [41]. It was suggested by these authors that the VES in the central nervous system could serve in an autoregulatory role. They also implicated vestibular efferents in this autoregulatory inner ear vestibular efferent coordination process, and we propose that this process is a major function of the inner ear macular system. Note that this process is identical to the so-called “fusimotor feedback system”, which is also specifically designed to “detect deviations from intended movement”, as outlined by Mathews [41]. 

Although this outline states that a conclusive functional role in mammals has yet to be confirmed [41], one can surmise why the existence of our proposed “slip and grip” emergency response process is crucial to maintain balance and prevent injury in the bipedal human. In short, there is close coordination between the vestibular and muscular systems. The proprioceptive system uses a simultaneous efferent-to-afferent process, enabling precise preplanning and immediate feedback of error. Muscle commands are caused by a decision made in the cerebral cortex. This peripheral efferent interaction occurs in the spinal cord to precisely predict the expected incoming afferent information from the Pacinian corpuscles of the affected joints, skin, and the Golgi tendon organs. For a particular movement, a muscle and the intrafusal muscle fibres of its own spindles are precisely and simultaneously coordinated with each other for this peripheral sensory process. The process is identical to the simultaneous inner ear vestibular efferent coordination process which is proposed here for the maculae of the inner ear (i.e., a feed forward command system). The substitution of vestibular reflexes by feedforward commands is likely to be a general principle for gaze and balance regulation during vertebrate, including human locomotion [81]. What are these feedforward commands, and how are they driven? One of our goals has been to suggest that the feedforward commands are driven by the otoliths.

As we have detailed in our proposal, vestibular efferent neuronal activity acts through the striated organelle, causing stereociliary movement in the type 1 vestibular hair cell. This precisely coincides with the otoconial displacement caused by the movement. It has been experimentally demonstrated that no vestibular afferent first-order axonal neural firing occurs (unless there is a discrepancy between the otoconial positioning instruction and the actual body positioning due to the efferent bodily motor instruction). Discrepancy is generated by an external factor such as an unexpected external sensory event, e.g., unrecognized uneven ground or an unexpected contact with an external object. Such an event initiates immediate vestibular afferent firing to draw attention to the crisis. The firing drives a motor correction and also a simultaneous vestibular efferent instruction to reposition the otolithic structures so that stability can be restored.

This vestibular efferent process differs from the well-recognized corollary discharge process. A corollary discharge is a copy of a motor command that is sent to the muscles to produce a movement. This copy or “corollary” does not produce any movement itself, but it is directed to other regions of the brain to inform them of the impending movement in order to reduce motor response discrepancies. Corollary discharge signals are important for the brain to identify sensory input arising from self-motion and to compensate for it. When a signal is sent from the motor cortex of the brain to the eye muscles (i.e., to generate an appropriate saccade), a copy of that signal (or efference copy) is sent through the brain as well. The brain does this in order to suppress midbrain visual processing during the saccade so that spurious visual input is suppressed during self-motion.

The complexity of the inner ear balance system has already been emphasized [37,38]. It is becoming possible to address this complexity now that we have some tests, and hopefully we can improve on them to be able to assess how the balance function of the macular system works. Development of vestibulospinal VEMPS at a clinical level would be expected to correlate with CDP, as present CVEMPs and OVEMPs have failed to do [82]. Use of this information combined with virtual reality physiotherapy and functional MRI investigations will increase our knowledge and improve therapeutic options and outcomes [83]. The technically challenging and highly time-consuming arduous electron microscopic tomography of more vestibular hair cells in the utricle will establish precise details of the connection between the stereociliary rootlets and the striated organelle associated with the various type I vestibular hair cell stereociliary arrays [84]. Similar electron microscopic tomography of the saccule needs to be undertaken and, based on the utricular findings, should show more evidence that the saccular STO maintains the apical cell structure of vestibular hair cells, which are more affected by gravity, for example, during the impact of walking. This information will lead to more effective diagnosis and therapy for vestibular disease. The potential exists for recognition of specific balance system diseases due to degeneration or malfunction in this process and for the development of specific focused treatment.

This paper has proposed a new concept for how the macular system of the inner ear works. Necessarily, it is a bilateral process with a central integration of information coming in from both sides [85]. In order to explain this theory, we have described an example of specific movements to characterize how the whole system works. The reader can think of their own examples of specific movement to address how this process works with any movement.

In conclusion, it is recognized that there is a strong interaction between various vestibular signals, an attenuation when appropriate, and an instantaneous integration of these signals with extravestibular cues. As outlined by McFadyen et al. [10], there is a need for a “separate internal feedforward model” or command system to modulate the final output of the vestibular signal, but, as yet, we do not understand how the brain accurately computes an estimate of body orientation and movement during active self-motion. Such a computation is necessary because “the ability to keep track of where we are going as we navigate through our environment requires knowledge of our ongoing location and orientation”, as stated by Carriot et al. [85]. The authors hope that this review will provide some insight into these complex processes.
